# Effect of Konjac Glucomannan on Structure, Physicochemical Properties, and In Vitro Digestibility of Yam Starch during Extrusion

**DOI:** 10.3390/foods13030463

**Published:** 2024-02-01

**Authors:** Mengshuang Hao, Xiaopei Zhu, Xiaolong Ji, Miaomiao Shi, Yizhe Yan

**Affiliations:** 1 College of Food and Bioengineering, Zhengzhou University of Light Industry, Zhengzhou 450001, China; haomengshuang1997@163.com (M.H.); zxiaopei0217@163.com (X.Z.); chengzi3090@126.com (M.S.); 2 Key Laboratory of Cold Chain Food Processing and Safety Control, Ministry of Education, Zhengzhou University of Light Industry, Zhengzhou 450001, China

**Keywords:** yam starch, konjac glucomannan, extrusion, structure, in vitro digestibility

## Abstract

In this study, the effect of konjac glucomannan (KGM, 0–5%) on the structure, physicochemical properties, and in vitro digestibility of extruded yam starch (EYS) was investigated. The EYS became rougher on the surface and the particle size increased as observed using scanning electron microscopy and particle size analysis. X-ray diffraction and Raman results revealed that the relative crystallinity (18.30% to 22.30%) of EYS increased, and the full width at half maxima at 480 cm^−1^ decreased with increasing KGM content, indicating the increment of long-range and short-range ordered structure. Differential scanning calorimetry and rheological results demonstrated that KGM enhanced thermal stability and the gel strength of EYS due to enhanced interaction between KGM and YS molecules. Additionally, a decrease in the swelling power and viscosity of EYS was observed with increased KGM content. The inclusion of KGM in the EYS increased the resistant starch content from 11.89% to 43.51%. This study provides a dual-modified method using extrusion and KGM for modified YS with high thermal stability, gel strength, and resistance to digestion.

## 1. Introduction

Yam, also known as Dioscorea, is a tuberous plant of the Dioscorea genus and the Dioscoreaceae family, with its main distribution areas being tropical and subtropical. According to reports, out of nearly 600 species of yam worldwide, 93 of them originate from China [1]. Yam was first recorded in the ancient Chinese book “Shennong Bencao Jing”, and has been cultivated and used in medicine for a long time [2]. As one of the important medicinal and edible foods in China, yam tubers contain abundant functional nutrients, such as starch, polysaccharides, saponins, etc., which contain a wide range of bioactivities and health benefits. For example, antioxidant, anti-inflammatory, antitumor, hypoglycemic, and lipid-lowering effects have been found in many studies [3]. Therefore, yam is widely used in traditional Chinese medicine, health foods, and daily diets.

Yam starch (YS) is the main constituent of fresh yam and its content accounts for 18%–25% of the fresh weight of the yam. YS is a smooth, uncracked, round or oval particle that varies in size from 15 to 30 μm. The amylose content of YS is high, and has good gelatinization characteristics, gel characteristics, and thermal stability [4]. In recent years, the modification of YS has become a research field that has attracted much attention. Methods such as wet grinding and drying treatment [5], hydrothermal treatment [6], and different drying treatments can influence the molecular structure, as well as physicochemical and functional properties, of YS.

Extrusion has been utilized to enhance the properties of starch and broaden its potential applications [7]. It involves mixing, blending, conveying, heating, kneading, and shaping, which is a high-temperature and short-time physical modification method [8]. During the extrusion process, the generated heat energy combined with the shear effect leads to changes in the physicochemical and digestibility of starch [9,10]. The gel stability, retrogradation [11], and freeze–thaw stability [12] of rice starch after extrusion were improved. Furthermore, extrusion under controlled conditions could decrease the digestibility of the starch [13,14,15]. Notably, although the use of extrusion in starch modification is well established, research on the impact of extrusion conditions on YS is relatively limited.

Hydrocolloid is a food additive that is often used to improve food quality. In addition, it has been used in studies on starch modification [16]. Recently, much attention has been paid to the study of interactions between non-starch polysaccharides and starch. The aim is to improve the functional properties and reduce the digestibility of starch [17]. Konjac gum, derived from the tuber of the konjac plant, is a high molecular weight polysaccharide, and its main component is konjac glucomannan (KGM) [18]. KGM is also a commonly used food additive with various properties such as hydrophilicity, thickening ability, emulsification, and gelation. At the same time, KGM has multiple health benefits, such as relieving constipation, facilitating weight loss, reducing the blood lipid index, and promoting the reproduction of healthy bacteria in the colon [19]. In addition, studies have shown that KGM exhibited a powerful thickening and stabilizing effect through hydrogen bonding interactions with starch, which inhibited the digestion rate of starch [20]. However, there is currently relatively limited research on the interaction of YS and KGM through extrusion, as well as the physicochemical and functional properties of the extrudates.

Therefore, in this study, we prepared YS-KGM extrudates with different concentrations of KGM (0%, 1%, 3%, and 5%; *w*/*w*) using a twin-screw extruder and investigated their particle morphology, crystalline structure, short-range ordered structure, rheology, thermal properties, and digestive characteristics in vitro. These results could help us to understand the effect of KGM on the structure, physicochemical properties, and in vitro digestibility of EYS. Meanwhile, it would provide a dual-modified method using extrusion and KGM for modified YS with high stability, gel strength, and low GI.

## 2. Materials and Methods

### 2.1. Materials

Chinese yam (*Dioscorea opposita* Thunb.) was sourced from Wen County, Henan Province, China. Konjac glucomannan (KGM) was provided by Shanghai Yuan Ye Biotechnology Co., Ltd. (Shanghai, China) with a viscosity of ≥15,000 mPa·s (80% purity). Pancreatin (8 × USP, P7545) was purchased from Sigma-Aldrich Trading Co., Ltd. (Shanghai, China), as was the amyloglucosidase (260 U/mL, A7095). The amylose assay kit was purchased from Solarbio Science and Technology Co., Ltd. (Beijing, China). The GOPOD assay kits used in this experiment were obtained from Megazyme International Ireland Ltd. (Wicklow, Ireland). All reagents were of analytical grade.

### 2.2. Extraction of Yam Starch (YS)

The extraction of YS was performed according to Jiang’s previous methods with appropriate modifications [21]. First, fresh Chinese yam was washed, peeled, cut into slices, and then beaten into a slurry after water was added. Subsequently, the homogenate was placed on a 100-mesh sieve and washed repeatedly with deionized water. After the filtrate settled for approximately 4 h, the supernatant was discarded. A 0.2% sodium hydroxide solution (4 L) was used to remove proteins. The precipitate was washed with deionized water until colorless, and with anhydrous ethanol (500 mL) to remove any remaining impurities. The washed precipitate was filtrated, dried, and sieved through a 100-mesh sieve to obtain YS.

### 2.3. Preparation of YS-KGM Extrudates

The YS-KGM extrudates were prepared with slight modifications based on our previous method [22]. Firstly, different contents of KGM (0%, 1%, 3%, and 5% *w*/*w*) were added to YS and thoroughly mixed using an electric stirrer. The moisture content was adjusted to 40% and the mixture was equilibrated under sealed conditions for 3 h. The mixture was then subjected to extrusion using a benchtop co-rotating twin-screw extruder (Process11, Thermo Fisher Scientific, Waltham, MA, USA). The diameter of the extrusion die was 5 mm and the diameter of the screw was 11 mm. During the extrusion process, different temperatures (40, 50, 60, 70, 80, and 90 °C) were set in different barrel sections, the sample feed rate was set at 2.5 g/min, and the screw speed was set at 180 rpm. After extrusion, the extrudates were dried at 40 °C for 24 h, subsequently pulverized using a laboratory scale grinder (1000A, Yongkang Red Sun electromechanical Co., Ltd., Yongkang, China), and sieved through a 100-mesh sieve. Finally, the YS-KGM extrudates were labeled as EYS, EYS-KGM-1%, EYS-KGM-3%, and EYS-KGM-5%, respectively.

### 2.4. Determination of Amylose Content

The amylose (AM) content was measured by an amylose assay kit (Solarbio Science and Technology Co., Ltd., Beijing, China). A 0.01 g sample (dry basis) was placed into a test tube, 1 mL of reagent I was added, and it was thoroughly mixed. After extraction in a water bath at 80 °C for 30 min, the supernatant was discarded by centrifugation at 3000× *g* at 25 °C for 5 min. The precipitate was shaken by adding 1 mL of ethyl ether for 5 min, centrifuged at 3000× *g* at 25 °C for 5 min, and the supernatant was discarded. A volume of 5 mL of reagent 4 was added to the precipitate, thoroughly dissolved, and bathed in water at 90 °C for 10 min. After cooling, the solution was centrifuged at 3000× *g* at 25 °C for 5 min, and the supernatant was taken to be tested. A total of 20 µL of the liquid to be measured, standard solution, and distilled water were placed in the measuring tube, standard tube, and blank tube, respectively. A total of 4 µL of reagent 5, 4 µL of reagent 6, and 172 µL of distilled water were added to each tube in order to mix well and then the absorption values were determined at 550 nm and 485 nm using a UV spectrophotometer (752, Shanghai Jinghua Technology Instrument Co., Ltd., Shanghai, China). The absorbance of the measurement tube, standard tube, and blank tube at 550 nm were denoted as *A_determination_*, *A_standard_* and *A_blank_*, respectively, those at 485 nm were denoted as *A’_determination_*, *A’_standard_* and *A’_blank_* respectively, and Δ*A_determination_* = (*A_determination_* − *A_blank_*) − (*A’_determination_* − *A’_blank_*) was calculated. Δ*A_standard_* = (*A_standard_* − *A_blank_*) − (*A’_standard_* − *A’_blank_*). Amylose content was calculated as follows:

$$Amylose\;content\;(mg/g)={\Delta A}_{determination}÷({\Delta A}_{standard}÷C)\times V÷W$$

where *C*: standard solution concentration, 0.4 mg/mL; *V*: 5 mL; *W*: sample quality, g.

### 2.5. Scanning Electron Microscopy (SEM)

An appropriate amount of sample was removed, and conductive adhesive was used to evenly lay it on the sample stage. To enhance conductivity, the sample was subjected to gold coating by sputter coating for 60 s. The sample was observed using a scanning electron microscope (Regulus 8100, Hitachi, Tokyo, Japan). The test was performed at 20 kV of voltage and pictures were taken at a magnification of 1000.

### 2.6. Particle Size Analysis

The suspension (3%, *w*/*v*) of samples was transferred to the sample dispersion pool of a laser particle size analyzer (LS13320/ULM2, Beckman Coulter Ltd., High Wycombe, UK). The appropriate obscuration level for instrument testing was used, typically ranging from 7% to 13%. D(4,3) represents the volume-averaged diameter, which was measured and recorded together with D10, D50, and D90.

### 2.7. X-ray Diffraction (XRD)

Before conducting the test, the sample needed to be balanced at room temperature for seven days with a saturated NaCl solution [23]. The crystalline structure of the sample was determined by an X-ray diffractometer (D8 Advance, Bruker, Karlsruhe, Germany). The testing conditions included a tube voltage of 40 kV, tube current of 2 mA, scanning speed of 4°/min, scanning range from 5° to 50°, sampling step size of 0.02°, continuous scanning mode, 1 repetition, and a confidence level of 99%. The relative crystallinity (RC) of the samples was calculated by the Jade 6.0 software.

### 2.8. Raman Spectroscopy

The starch sample was placed in the cap of a centrifuge tube and pressed flat to make its surface smooth. The sample was measured using a portable Raman spectroscopy system (BWS465-785S, B&W Tek, Plainsboro Township, NJ, USA), and the full width at half maxima (FWHM) of the characteristic peak near 480 cm^−1^ was calculated using software to determine the short-range ordered structure of starch.

### 2.9. Differential Scanning Calorimetry (DSC)

Thermal properties of YS-KGM extrudates were measured by differential scanning calorimeter (Q20, TA Instruments, New Castle, DE, USA). A total of 3 mg (dry basis) of the sample was weighed and placed in an aluminum pan. After that, the deionized water (1:3, *w*/*w*) was added, sealed, and equilibrated for 12 h. Scanning was performed at a rate of 10 °C/min over the range of 30 to 120 °C. During the test, an empty aluminum pan was used as a reference. Data analysis was performed using TA Instruments Universal Analysis 2000 software.

### 2.10. Rapid Viscosity Analyzer (RVA)

A total of 3 g (dry basis) of the sample was weighed and placed in an aluminum canister with deionized water for a total weight of 28 g. Following the standard program 1, the sample was tested using a Rapid Viscosity Analyzer (RVA) (RVA4500, Perten Instruments, Hagersten, Germany). The RVA TCW3 software was used to obtain the gelatinization curve and corresponding parameters.

### 2.11. Dynamic Rheological Analysis

The dynamic rheological properties of YS-KGM extrudates were studied using a rotational rheometer (Discovery HR-1, TA instrument Inc., Newcastle, DE, USA). The sample gels obtained from RVA were transferred to the rheometer plate. Silicone oil was applied to the edges of the sample, which helped to maintain gel integrity and consistency during rheological measurements. The rheological test was conducted at 25 °C. Frequency sweeps in the range of 0.1–20 Hz were performed at a strain of 1%. The storage modulus and loss modulus were recorded to evaluate the rheological properties of the sample gels.

### 2.12. Solubility and Swelling Power

The sample (0.5 g, dry basis, *W*_0_) and deionized water (25 mL) were thoroughly mixed in a centrifuge tube. The mixture was then heated in a 90 °C water bath for 30 min, cooled to room temperature, and centrifuged (5000 r/min, 20 min). The supernatant was collected and dried to constant weight, denoted as *W*_1_ (g), and the weight of the precipitate in the centrifuge tube was noted as *W*_2_ (g). The solubility (*S*) and swelling power (*SP*) were calculated by the following formulas:

$$S\left(\%\right)=\frac{W_{1}}{W_{0}}\times 100$$

$$SP(g/g)=\frac{W_{2}}{W_{0}(1-S/100)}$$

### 2.13. In Vitro Digestion

The in vitro digestion of the sample was performed with slight modifications based on the method described by our group [24]. A total of 4 mL of 0.1 mol/L sodium acetate buffer solution was added to the weighed sample (200 mg, dry basis). After mixing well, the samples were gelatinized in a shaker (100 °C, 30 min) and hydrolyzed by adding 1 mL of the mixed enzymes. A volume of 0.1 mL of the digestible solution was taken out at 20 and 120 min, respectively, and 4 mL of 70% ethanol was mixed thoroughly to inactivate the enzyme. After centrifugation at 5000 r/min for 10 min, 0.1 mL of the supernatant was mixed with 3 mL of GOPOD and then the color was developed in a 45 °C water bath for 20 min. The absorbance was tested with a UV spectrophotometer at 510 nm. Similarly, 0.1 mL of standard glucose solution and 0.1 mL of deionized water were taken as standard and blank control. The digestion characteristics of the YS-KGM extrudates were determined by calculating the contents of rapidly digestible starch (*RDS*), slowly digestible starch (*SDS*), and resistant starch (*RS*).

$$RDS\left(\%\right)={(G}_{20}-FG)\times 0.9$$

$$SDS\left(\%\right)={(G}_{120}-G_{20})\times 0.9$$

$$RS\left(\%\right)=1-RDS-SDS$$

where the content of glucose at 20 or 120 min of hydrolysis is denoted by *G*_20_ or *G*_120_; the content of glucose in the sample before hydrolysis is expressed as *FG*.

### 2.14. Statistical Analysis

The data collection for all experiments consisted of three parallel experiments, and the data were presented as mean ± standard deviation. Significance analysis was performed using the Duncan test in SPSS Statistics 26.0 software (IBM Inc., Chicago, IL, USA) (*p* < 0.05). SPSS software was also used for the Pearson correlation analysis. Graphs were generated using Origin 2022 (Origin Lab Inc., Northampton, MA, USA) software in this study.

## 3. Results and Discussion

### 3.1. Amylose Content

Starch particles contain amylose and amylopectin, and amylose accounts for 5–35% of most native starches, which has a significant impact on starch properties in food. Table 1 shows that the amylose (AM) content was 26.49% for YS and 27.72% for EYS. The AM content of EYS was higher than that of YS, which indicated that extrusion treatment increased the AM content of YS. This might be due to the molecular chain degradation of starch after extrusion [25].

### 3.2. Particle Morphology and Size Distribution

**Figure 1:**
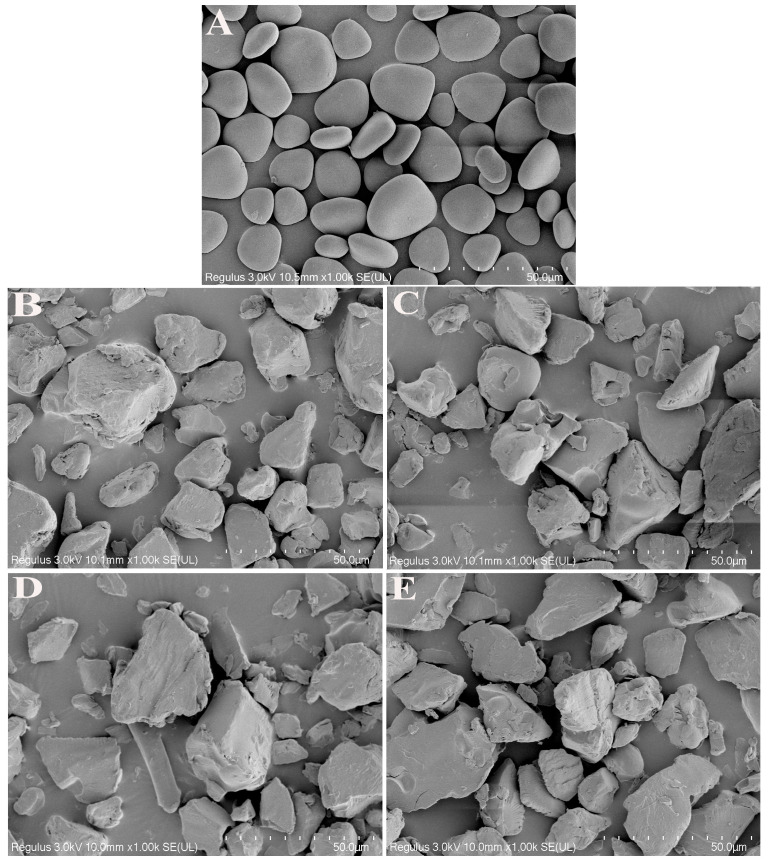
SEM images of YS, EYS, and EYS-KGM. (**A**) YS, (**B**) EYS, (**C**) EYS-KGM-1%, (**D**) EYS-KGM-3%, (**E**) EYS-KGM-5%. YS, yam starch. EYS, extruded YS. KGM, konjac glucomannan.

Figure 1A–E show the SEM images of YS, EYS, and EYS-KGM. YS appeared as an elliptical particle with a smooth and crack-free surface. In comparison to native YS, the surface roughness of all extruded samples increased and exhibited an irregular rock-like shape [26]. As the KGM content increased, the large particle content of EYS also increased. This might be because the extrusion caused the molecular rearrangement of starch, creating a structure in which KGM aggregated with starch molecules. In addition, previous studies have indicated that extrusion-treated KGM particles had a larger particle size than untreated ones [27].

**Figure 2:**
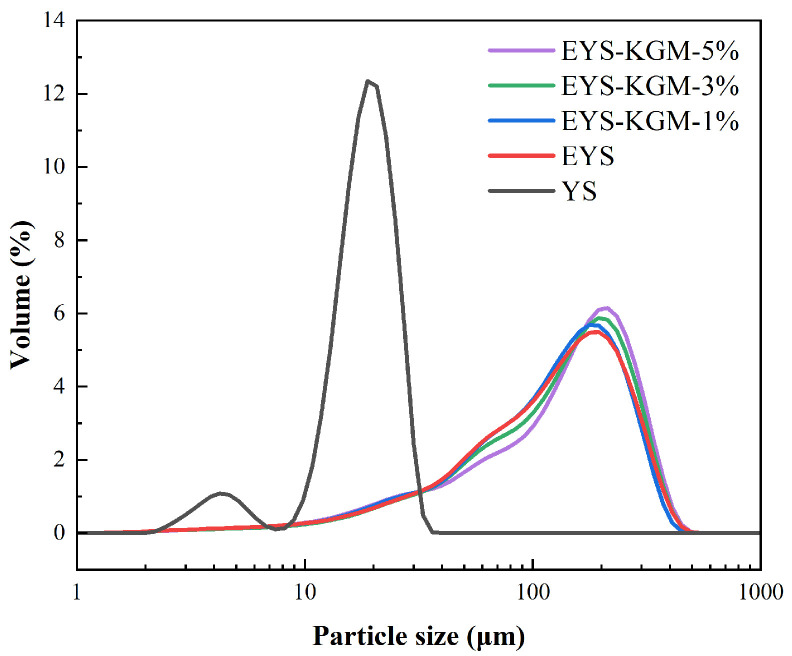
Particle size distribution curves of YS, EYS, and EYS-KGM. YS, yam starch. EYS, extruded YS. KGM, konjac glucomannan.

The particle size distributions of YS, EYS, and EYS-KGM are shown in Figure 2. The particle sizes of native YS were mainly concentrated in two ranges of 2–7 μm and 10–30 μm. The number of large particles increased in the sample after the extrusion treatment. The particle size of the peak volume fraction tended to move toward larger sizes when more KGM was added. In the description of particle size distribution, the D-value is the most commonly used parameter, which is used to represent the intercept of cumulative mass percentage [28]. From Table 1, the extruded YS have higher values of D(4,3), D90, D50, and D10 than those of native YS.

### 3.3. Crystalline Structure

**Figure 3:**
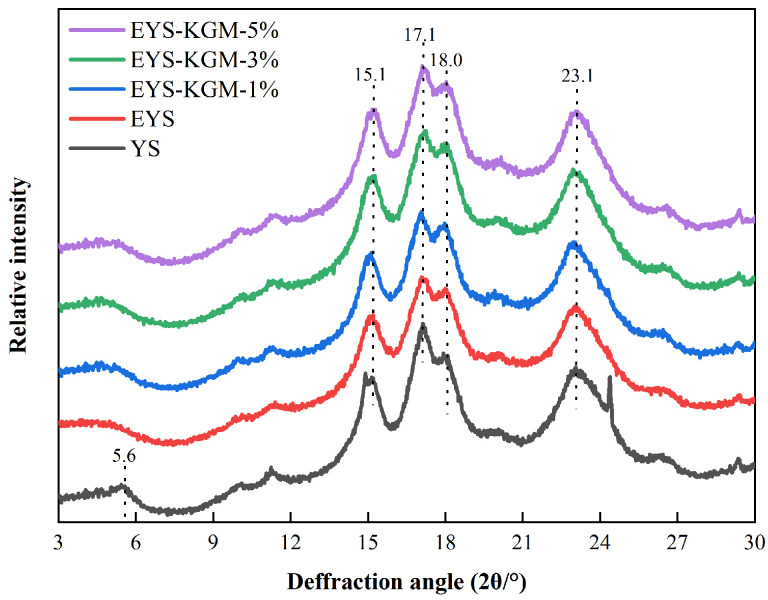
XRD patterns of YS, EYS, and EYS-KGM. YS, yam starch. EYS, extruded YS. KGM, konjac glucomannan.

Figure 3 displays the XRD patterns of YS, EYS, and EYS-KGM. Generally, the crystal types of starch are divided into A, B, and C types. Starches that show strong double peaks at about 17° and 18° can be classified as type A, while those that have a prominent peak at about 5.6° and a wide peak at about 23° are considered type B. The type C is a hybrid of type A and type B; it has broad peaks at approximately 17° and 23°, with some minor peaks around 5.6° and 15° [29]. YS showed a typical C-type crystalline structure because its diffraction peaks were located at 5.6°, 15.1°, 17.1°, 18.0°, and 23.1° [30]. After the extrusion treatment, the diffraction peak at 5.6° disappeared. The diffraction peaks of EYS were located at 15.1°, 17.1°, 18.0°, and 23.1°, exhibiting an A-type structure. The relative crystallinity (RC) of EYS decreased from 23.37% to 18.30% compared to YS, which was due to the fact that extrusion destroyed the natural arrangement of starch molecules and transformed it into an amorphous structure [31]. Furthermore, with the increase of KGM, the RC of EYS-KGM-1%, EYS-KGM-3%, and EYS-KGM-5% increased to 20.10%, 21.07%, and 22.30%, respectively, which is in line with Ning’s findings [20]. The interaction between KGM and branched starch chains led to the rearrangement of starch helical structures and an improvement in starch recrystallization [32].

### 3.4. Short-Range Ordered Structure

**Figure 4:**
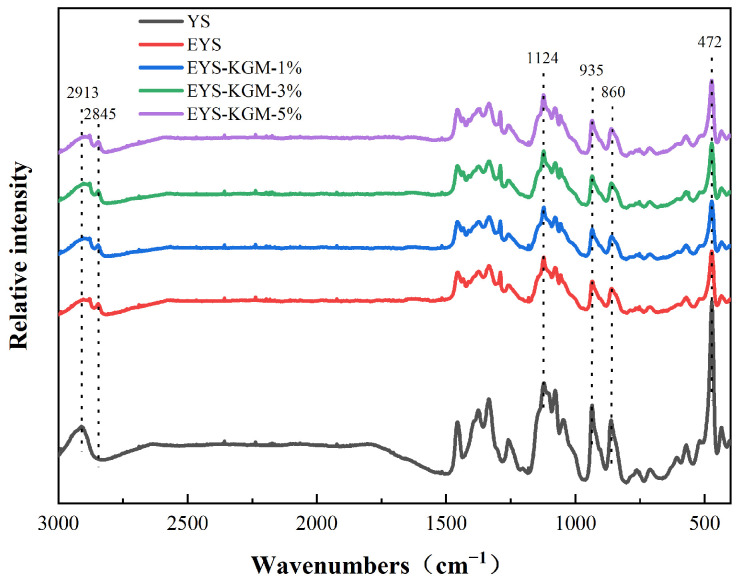
Raman spectra of YS, EYS, and EYS-KGM. YS, yam starch. EYS, extruded YS. KGM, konjac glucomannan.

The Raman spectra are shown in Figure 4; the peak intensity of YS became weaker after extrusion treatment. The spectral band at 2800~3000 cm^−1^ belonged to the C-H stretching vibration [33]. The spectral band at 800~1500 cm^−1^ was the fingerprint region of YS. The strong spectral band at 472 cm^−1^ corresponded to the backbone vibration region of the cyclic ring of pyranose in YS, and the FWHM of the absorption peak here could be used for characterizing the short-range ordering of the YS. In general, as the short-range order increased, the FWHM at 472 cm^−1^ decreased [34,35]. From Table 1, the FWHM of extruded YS was remarkably higher than that of the native one, which indicated that the extrusion resulted in a reduction of the double helix structure of YS and a decrease in its short-range orderliness. In addition, compared to EYS, the FWHM of EYS-KGM decreased with the increase of KGM content, resulting from an enhanced hydrogen bond of KGM with YS molecules.

### 3.5. Thermal Properties

**Figure 5:**
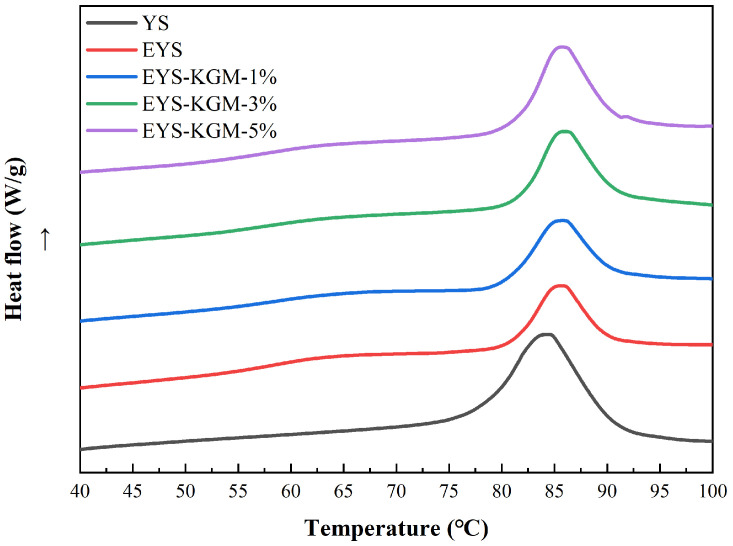
DSC curves of YS, EYS, and EYS-KGM. YS, yam starch. EYS, extruded YS. KGM, konjac glucomannan.

As shown in Figure 5, all starch samples showed typical starch gelatinization peaks, and the specific gelatinization parameters are listed in Table 2. It can be observed that extrusion increased the gelatinization temperature of the starch. This may be due to the fact that extrusion treatment enhanced the interaction of the amylose with either the amylose or the amylopectin, which inhibited the granule swelling and delayed the gelatinization [36]. Additionally, a reduction in the double helical structure after extrusion treatment also led to a lower enthalpy (ΔH) of gelatinization. When KGM was added from 0% to 5%, EYS-KGM-5% gave the highest gelatinization temperature (T_O_, T_P_, T_C_). In general, KGM causes an increase in parameters (T_O_, T_P_, T_C_) [37]. Moreover, extrusion and KGM treatment increased the gelatinization temperature range (T_c_–T_O_) from 12.21 to 14.41 °C. Compared to EYS, the EYS-KGM-1%, 3% and 5% showed a gradual increase in ΔH from 6.92 J/g to 9.60 J/g. This was due to the crystalline and helix structure enhanced with increasing KGM content [32]. Previous research has indicated that KGM could increase the gelatinization temperature and ΔH of starch [38].

### 3.6. Solubility and Swelling Power

The S and SP of YS, EYS, and EYS-KGM are shown in Table 2. Generally, the S and SP can reflect the damage degree of starch granules during processing, such as crushing, cooking, and microwaving [39]. According to the results shown in Table 2, extrusion increased S and decreased the SP of YS because extrusion destroyed the starch structure and led to the depolymerization and reorganization of starch molecules [40]. In addition, SP of EYS-KGM decreased with increasing KGM content, which was because extrusion may lead to rearrangement of starch molecules with KGM and form a more rigid structure, limiting the swelling of starch granules [41].

### 3.7. Pasting Properties

**Figure 6:**
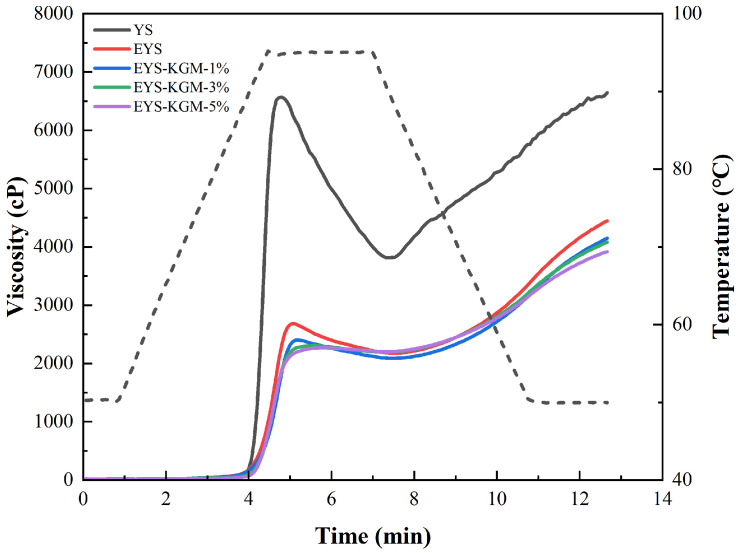
RVA curves of YS, EYS, and EYS-KGM. YS, yam starch. EYS, extruded YS. KGM, konjac glucomannan.

Figure 6 and Table 2 exhibit the pasting properties of YS, EYS, and EYS-KGM. The peak viscosity (PV) indicates the equilibrium point when starch granules absorb water and swell, and the breakdown viscosity (BD) is a reflection of the shear resistance of starch at high temperatures while final and setback viscosity (FV and SB) are related to the short-term retrogradation of amylose [42]. Compared to native YS, PV, BD, FV, and SB of extruded YS samples were significantly reduced. The research showed that the gelatinization property of starch was closely related to the particle size and swelling degree of starch particles [43]. During extrusion, starch pasting and degradation occurred, starch granules lost their integrity, and their swelling degree decreased, resulting in viscosity loss [36]. When the addition of KGM increased, the PV of EYS-KGM gradually decreased from 2614 to 2297 cP, indicating better mobility, which was because KGM could smooth the flow and inhibit the abrasion of starch granules. In addition, KGM could prevent the swelling of starch granules, thereby reducing PV [44]. The BD of EYS-KGM also gradually decreased from 551 to 43 cP with increasing the addition of KGM due to higher stability during heating and stirring. As the KGM addition increased, the SB of EYS-KGM also reduced from 2216 to 1676 cP because a stronger interaction between amylose and more KGM inhabited the short-term retrogradation of amylose [45]. 

### 3.8. Rheological Properties

**Figure 7:**
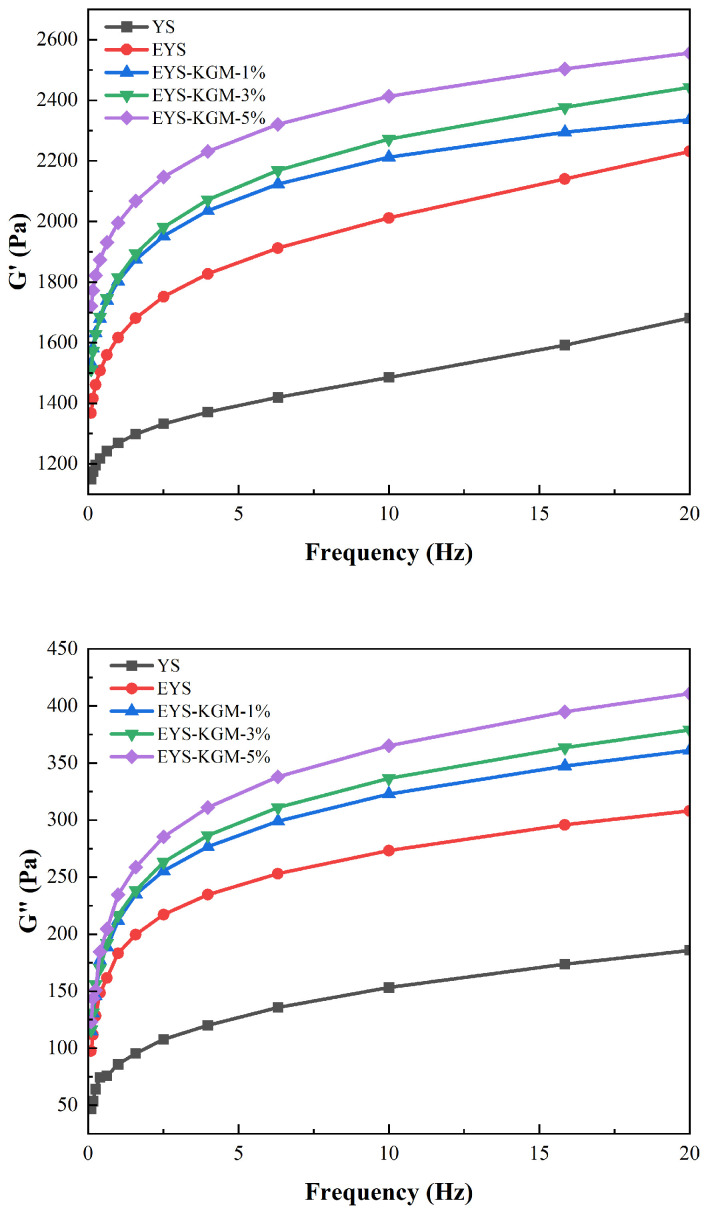
Rheological curves of YS, EYS, and EYS-KGM. YS, yam starch. EYS, extruded YS. KGM, konjac glucomannan.

Figure 7 demonstrates the rheological properties of YS, EYS, and EYS-KGM. The storage modulus (G′) and loss modulus (G″) are respectively related to the elastic and viscous properties of the sample [46]. From Figure 6, compared to native YS, extruded YS samples showed a significant increase in both G′ and G″, which indicated that the elasticity and viscosity of YS gels gradually increased with the increase of KGM content. This could be a result of the reorganization of amylose molecules that have been released through the pores and cracks on the surface of the starch granules when mixed with KGM during the extrusion process. This reorganization increased the stiffness and formed a stronger gel network structure [47].

### 3.9. In Vitro Digestibility

**Figure 8:**
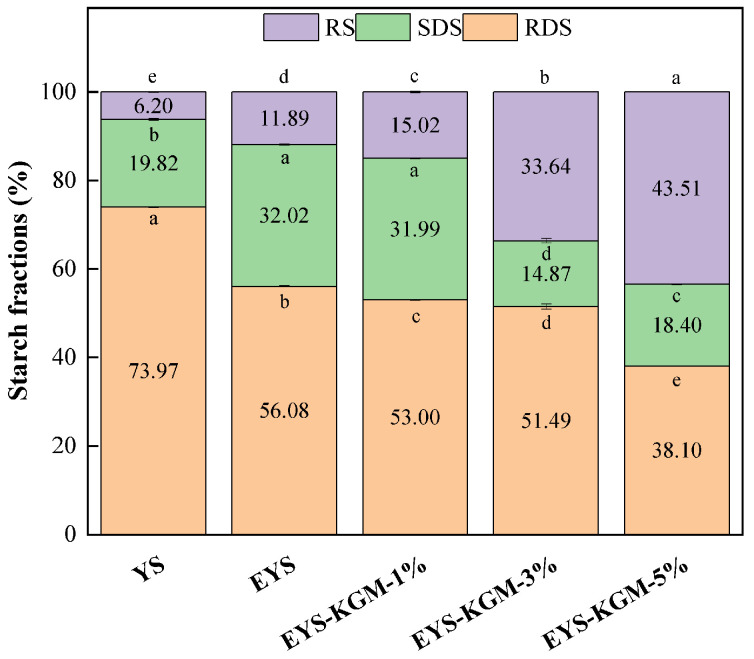
RDS, SDS, and RS contents of YS, EYS, and EYS-KGM. YS, yam starch. EYS, extruded YS. KGM, konjac glucomannan. Different letters for RDS, SDS or RS indicate a significant difference (*p* < 0.05).

Figure 8 illustrates the in vitro digestion results of YS, EYS, and EYS-KGM. After extrusion, the RDS content of YS decreased from 73.97% to 56.08%, while its RS content increased from 6.20% to 11.89%. This observed change could be attributed to the disruption of starch particles caused by extrusion, resulting in the development of a more densely packed structure due to increased interactions between starch molecules [48]. In addition, compared with EYS, as the amount of KGM increased, the in vitro digestion of yam starch was inhibited. As evidenced by a decrease in RDS content from 56.80% to 38.10%, the RS content of EYS-KGM increased from 11.89% to 43.51%. This could be since KGM molecules wrapped around the starch molecules, reducing the contact sites between digestive enzymes and starch, resulting in reduced digestibility of YS [17]. There was an increase in the SDS content for EYS and EYS-KGM-1% compared to YS. However, when the KGM content was increased to 3% or 5%, there was a decrease in the SDS content. In the extruded samples, the overall contents of SDS and RS were increased, and with the increase of KGM content, SDS was transformed into RS. Similar conclusions have been drawn that KGM can act as a barrier to reduce starch digestibility and increase the RS content [49]. At the same time, the hydration properties of KGM could improve starch concentration, thereby slowing down the coagulation and digestibility of starch [50]. In general, starches with high RS content can be used in foods with low GI.

### 3.10. Pearson Correlation Analysis between KGM Content and Indexes of EYS-KGM 

**Figure 9:**
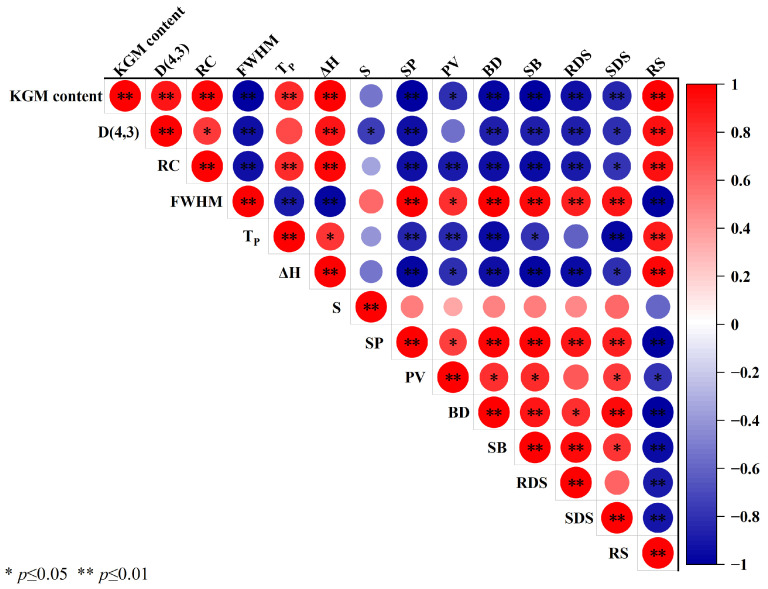
Pearson correlation analysis between KGM content and indexes of EYS-KGM. YS, yam starch. EYS, extruded YS. KGM, konjac glucomannan.

Figure 9 shows the correlation analysis results between the KGM content and indexes of EYS-KGM. KGM content was significantly positively correlated with D(4,3), RC, T_P_, ΔH, and RS content, and significantly negatively correlated with FWHM, SP, PV, BD, RDS content, and SDS content. Meanwhile, in terms of digestibility, there was a significant positive correlation between RS content and KGM content, D(4,3), RC, T_P_, ΔH, and a significant negative correlation between RS content and FWHM, SP, BD, SB. Thus, we can conclude that the addition of more KGM improved the long-range and short-range structure of EYS, ultimately increased thermal stability and gel strength, and reduced the accessibility between the digestive enzymes and starch, leading to a decrease in starch digestibility.

## 4. Conclusions

In summary, the effect of KGM (0%, 1%, 3%, and 5%) on the structure, and properties of EYS was investigated. The surface of the EYS-KGM particles became rough and the particle size increased. The extruded crystals showed an A-type structure. Meanwhile, as the KGM content increased, there was a noticeable rise in the relative crystallinity and short-range order of EYS molecules. In addition, the increased addition of KGM during extrusion led to an increase in the thermal stability, gel structure, and RS content of EYS-KGM while reducing their reduced viscosity, swelling, and RDS content. Furthermore, Pearson correlation analysis showed that KGM adding content was closely correlated with the structure, physicochemical, and digestive properties of EYS-KGM. Therefore, this research offers a novel method for producing functional YS-based food by combining twin-screw extrusion with KGM.

## Figures and Tables

**Table 1:** AM content, particle size distribution, RC, and FWHM of YS, EYS, and EYS-KGM.

Samples	YS	EYS	EYS-KGM-1%	EYS-KGM-3%	EYS-KGM-5%
AM (%)	26.49 ± 0.44 ^b^	27.72 ± 0.32 ^a^	-	-	-
D(4,3) (μm)	18.94 ± 0.00 ^e^	148.62 ± 0.47 ^c^	144.33 ± 0.93 ^d^	154.21 ± 1.31 ^b^	159.21 ± 2.21 ^a^
D10 (μm)	11.28 ± 0.00 ^d^	31.65 ± 0.75 ^ab^	30.31 ± 0.89 ^bc^	32.52 ± 1.16 ^a^	29.38 ± 1.27 ^c^
D50 (μm)	19.23 ± 0.00 ^d^	135.99 ± 0.47 ^c^	134.04 ± 1.03 ^c^	144.84 ± 1.32 ^b^	153.02 ± 2.21 ^a^
D90 (μm)	26.97 ± 0.00 ^e^	284.66 ± 0.29 ^c^	273.73 ± 0.99 ^d^	290.89 ± 1.67 ^b^	298.84 ± 2.74 ^a^
RC (%)	23.37 ± 0.15 ^a^	18.30 ± 0.20 ^e^	20.10 ± 0.10 ^d^	21.07 ± 0.21 ^c^	22.30 ± 0.10 ^b^
FWHM (cm^−1^)	17.13 ± 0.09 ^e^	18.80 ± 0.02 ^a^	18.70 ± 0.05 ^b^	18.47 ± 0.03 ^c^	18.35 ± 0.05 ^d^

Values are expressed as the mean ± SD of three measurements. In the same row, different lowercase letters indicate a significant difference (*p* < 0.05). AM, amylose. D(4,3) represents the volume average particle size, and D10, D50, and D90 are the particle sizes at 10%, 50%, and 90% of the volume of all particles, respectively. RC, relative crystallinity. FWHM, the full width at half maxima. The “-“ indicates untested.

**Table 2:** Thermal properties, S, SP, and pasting properties of YS, EYS, and EYS-KGM.

Samples	YS	EYS	EYS-KGM-1%	EYS-KGM-3%	EYS-KGM-5%
T_O_ (℃)	78.26 ± 0.08 ^d^	81.21 ± 0.07 ^b^	80.73 ± 0.02 ^c^	81.78 ± 0.10 ^a^	81.67 ± 0.07 ^a^
T_P_ (℃)	84.51 ± 0.04 ^d^	85.42 ± 0.15 ^c^	85.56 ± 0.21 ^bc^	85.84 ± 0.03 ^a^	85.70 ± 0.08 ^ab^
T_C_ (℃)	94.33 ± 0.29 ^c^	93.42 ± 0.10 ^d^	94.90 ± 0.53 ^b^	95.05 ± 0.03 ^b^	96.08 ± 0.30 ^a^
ΔH (J/g)	14.92 ± 0.22 ^a^	6.92 ± 0.20 ^e^	7.52 ± 0.25 ^d^	8.50 ± 0.05 ^c^	9.60 ± 0.18 ^b^
S (%)	10.80 ± 0.00 ^b^	20.09 ± 0.68 ^a^	20.79 ± 0.54 ^a^	19.88 ± 0.42 ^a^	19.65 ± 0.14 ^a^
SP (g/g)	16.25 ± 0.10 ^a^	12.72 ± 0.09 ^b^	12.57 ± 0.07 ^b^	11.93 ± 0.12 ^c^	11.42 ± 0.01 ^d^
PV (cP)	6569 ± 1 ^a^	2614 ± 100 ^b^	2394 ± 10 ^c^	2292 ± 23 ^d^	2297 ± 2 ^d^
BD (cP)	2698 ± 80 ^a^	551 ± 63 ^b^	453 ± 11 ^c^	116 ± 3 ^d^	43 ± 4 ^e^
FV (cP)	6645 ± 4 ^a^	4279 ± 240 ^b^	4087 ± 89 ^bc^	4095 ± 22 ^bc^	3880 ± 81 ^c^
SB (cP)	2774 ± 86 ^a^	2216 ± 78 ^b^	2079 ± 25 ^bc^	1920 ± 42 ^cd^	1676 ± 12 ^e^

Values are expressed as means ± SD of three measurements. In the same row, different lowercase letters indicate a significant difference (*p* < 0.05). YS, yam starch. EYS, extruded YS. KGM, konjac glucomannan. T_O_, T_P_ and, T_C_ represent the onset, peak, and conclusion temperature. ΔH, gelatinization enthalpy. S, solubility. SP, swelling power. PV, peak viscosity. BD, breakdown viscosity. FV, final viscosity. SB, setback viscosity.

## Data Availability

The data presented in this study are available on request from the corresponding author. The data are not publicly available due to privacy restrictions.
